# Synthesis and Characterization of Mesoporous Materials Functionalized with Phosphinic Acid Ligand and Their Capability to Remove Cd(II)

**DOI:** 10.3390/molecules29215199

**Published:** 2024-11-02

**Authors:** Khayra Mersellem, Djamila Bouazza, Irene Malpartida, Pedro Maireles-Torres, Anne Boos, Hary Demey, Hafida Miloudi

**Affiliations:** 1 Laboratory of Applied Organic Synthesis, University of Oran1, B.P 1524 El M’naouer, Oran 31000, Algeria; kmersellem@yahoo.com; 2 Laboratory of Materials Chemistry, University of Oran1, B.P 1524 El M’naouer, Oran 31000, Algeria; bouaza_dj@yahoo.fr; 3 Deasyl, S.A., Plan-les-Ouates, 1228 Geneva, Switzerland; irene.malpartida@gmail.com; 4 Department of Inorganic Chemistry, Crystallography and Mineralogy, Faculty of Science, University of Malaga, Campus de Teatinos, 29071 Malaga, Spain; maireles@uma.es; 5 Laboratory for Recognition and Molecular Separation Processes Hubert Curien Multidisciplinary Institute, 67000 Strasbourg, France; anne.boos@unistra.fr; 6 CEA, LITEN, DTCH, Laboratoire Réacteurs et Procédés (LRP), Grenoble-Alpes University, F-38000 Grenoble, France

**Keywords:** cadmium, DIOPA, MCM-41, sorption

## Abstract

This article presents a study of cadmium removal from nitrate medium using adsorption in calcined mesoporous silica (MCM-C), mesoporous silica doped (MCM_DIOPA), and calcined and impregnated mesoporous silica (MCM@DIOPA), with diisooctylphosphinic acid (DIOPA). The sorbents were synthesized via a sol–gel method. Several characterization techniques, such as XRD, FTIR spectroscopy, N_2_ sorption and elemental analysis, have been used to determine the main structural, textural, and chemical properties of prepared sorbents. Batch adsorption and kinetics tests were carried out, where the influence of pH and contact time of the sorbents and their role in cation removal were studied. Experimental results show poor sorption efficiencies with MCM-C and MCM_DIOPA at pH 5.85. At the same pH, better cadmium extraction was attained by MCM@DIOPA and was achieved within 30 min. The pseudo-second-order model is the most appropriate model to describe the elimination mechanism of Cd(II) ions. The Langmuir equation was used to model the sorption isotherm and the maximum sorption capacity of Cd(II) is 22.16 mg/g (200 mmol/kg). The complex type of the probable extracted species isCdL2-HL.

## 1. Introduction

The contamination of aquatic and terrestrial ecosystems by toxic heavy metals, such as Cr(VI), Hg(II), Pb(II), etc., constitutes an environmental problem [1,2]. Their release and dispersion in the environment has increased with the intensive development of industry and human activities. Most of them are non-degradable and persistent. Among these hazardous metallic species, nickel (Ni^2+^), lead (Pb^2+^), zinc (Zn^2+^), copper (Cu^2+^), cadmium (Cd^2+^), chromium (Cr^3+^) and mercury (Hg^2+^) are the most common water pollutants with extreme toxicity, as they are harmful to the environment and human beings even at low concentrations and can have dangerous impactsasthey accumulate in organisms [1,3,4,5,6], which is a serious concern for public safety and health [7,8]. According to the World Health Organization (WHO), certain quantities of heavy metals are tolerated in drinking water, for example: nickel (20 µg/L), Pb (10 µg/L), copper (2000 µg/L), cadmium (5 µg/L), total chromium (50 µg/L), and total mercury (1 µg/L). Contrary to many organic pollutants, heavy metals are generally refractory, and their adsorption from solutions, or soils, or using biological decontamination isa hard task. Therefore, heavy metals contaminate waters (surface or ground) and surrounding soils [9,10,11].

Cadmium is a non-nutritive metal considered harmful to the environment and to hu-mans. It can enter the soil, water, and air through activities such as agriculture (phos-phatefertilisers, pesticides), industry (mining, textiles, metallurgy, electroplating, incin-eration of solid and liquid fuels, welding, batteries, etc.), inappropriate storage or combus-tion of cadmium-rich waste, smoking, etc. [12,13,14,15]. In the environment, Cd exists only in a single oxidation state (II), it dissolves quickly in acidic leachates, and thus pollutes fresh water used for human consumption [16,17]. Cadmium can affect certain organs of the human body given its high solubility and its non-biodegradable nature [11,12,18].

In order to minimize the harmful effects of cadmium contained in water and soil, research has been undertaken. For the elimination of heavy metals contained in industrial waste (liquid or solid), different techniques have been used [14,19,20,21,22]. Liquid–solid extraction remains the most suitable method for the removal and separation of various contaminants, due to the availability of low-cost materials, simplicity of the processing system, and its high efficiency [23]. Among the adsorbents used in liquid–solid extraction, mesoporous materials present advantages due to their large specific surface area, high pore volume and perfectly controlled porosity, as well as their mechanical and thermal stabilities [24,25]. However, MCM-41 silica, with its characteristic properties, has been used effectively in different applications for catalysis [26], adsorption of heavy metal [24,27], CO_2_ capture [28], dyes sorption [29], drug delivery [30].

The functionalization of materials with selective functional groups can improve their physico-chemical properties and their sorption performances. The common functionalized sorbents found in the literature are: Zeolites [31], activated carbon [32,33], organoclay [34,35,36,37,38,39], nanoparticles [40], and phosphate solids [41,42,43]. The impregnation of MCM-41 silica by acidic organic groups is a great functionalization method used to improve its metal selectivity and separation [44,45,46,47]. Organphosphorus ligands are the most organic acids that have found extensive usage in solvent extraction [48,49,50,51].

Many other materials impregnated by organophosphorus extractants have been the scope of previous well-documented studies [35,44,46,52,53,54,55]. Unlike certain organophosphorus acids, the diisooctylphosphinic acid (DIOPA) ligand has been used very little to extract metals [56,57,58,59].

The present work investigates, forthe first time, the removal of Cd(II) ions from aqueous solutions using a MCM-41 silica functionalized by DIOPA. For this purpose, a series of materials were prepared and used for the elimination of Cd in an aqueous solution. The obtained results were correlated between the nature of functionalization used and the obtained capture of the Cd species.

## 2. Materials and Methods

### 2.1. Materials

The reagents used for the preparation of silica sorbent were: tetraethoxysilane (TEOS 98%), as a silicon source, cetyltrimethylammonium bromide (CTAB, 98%), as a structure-directing agent, and methanol (CH_3_OH, 99.6%), which were provided by Sigma Aldrich (St. Louis, MO, USA),and sodium hydroxide (NaOH, 99%), which was providedby Biochem.DIOPA (purity > 95%) was supplied by Fluka (Seelze, Germany) and used without further purification, and chloroform (CHCl_3_, 99% purity) from VWRchemicals (Barcelona, Spain) andnitric acid (HNO_3_, 96%) were purchased from Sigma Aldrich.

Cadmium solutions were prepared by dissolving Cd(NO_3_)_2_·4H_2_O salt, from Sigma Aldrich (98%), in demineralized water. In this study, the ionic strength of the solution was maintained at a constant value (0.1 M) by adding nitrate salt (NaNO_3_ 99–105%), which came from Sigma Aldrich. The pH was adjusted using dilute solutions of HNO_3_ or NaOH (as required).

### 2.2. Preparation of Sorbents

Mesoporous silica was synthesized following the classic method proposed by Firouzi et al. [60], which was adapted later by Boos et al. [61]. The MCM-41 material was prepared by dissolving an amount of 65,601 g of CTAB in 252 mL of NaOH solution (0.1 M) and stirring for 4 h at 60 °C. Then, 416,546 g of methanol was added to the previous mixture under continuous stirring for 4 h. Next, 20,833 g of TEOS was added under vigorous stirring for 1 h at 60 °C and then at 25 °C for 24 h. Finally, the resulting solid was filtered, dried and calcined at 550 °C for 10 h to eliminate CTAB and to release the pores. The obtained material is denoted as MCM-C.

#### 2.2.1. Preparation of the Impregnated Sorbent

**Figure 1:**
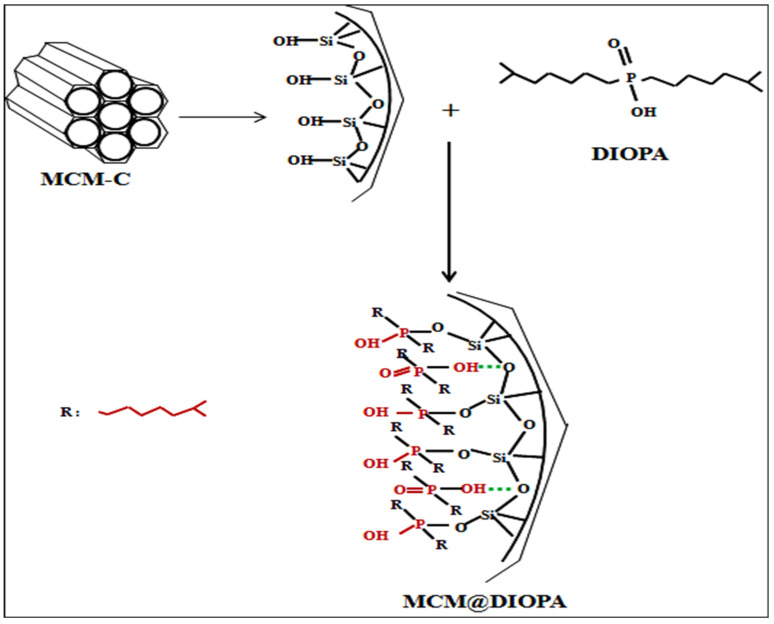
Interaction mechanism between DIOPA and MCM-C.

DIOPA was introduced into the MCM-C support using the dry impregnation method [53,55]. An appropriate amount of MCM-C (1 g) was mixed with a high purity solution of chloroform (25 mL) containing 0.7 mmol of DIOPA. The mixture was stirred under atmospheric pressure until total solvent evaporation. The obtained solid was washed with 0.1 M HNO_3_ solution to avoid releasing the extractant molecule during the adsorption study, and then dried in an oven at 60° C for 24 h. This sample was denoted as MCM@DIOPA.The interaction of the ligand with the silicic matrix can be carried out as shown in Figure 1.

#### 2.2.2. Preparation of the Doped Sorbent

Doped MCM-41 silica was prepared using a sol–gel process following the method proposed by Boos et al. [61]. The ligand was added at the same time as methanol. The solid obtained was washed with distilled water until neutralization and dried at 60 °C for 24 h. The obtained solid is denoted as MCM_DIOPA.

### 2.3. Adsorbent Characterization

The crystalline phases were identified by X-ray diffraction (XRD) using a Bruker (Leipzig, Germany) D8 Advance diffractometer with Cu Kα radiation (λ  =  1.5406 Å) over time in 0.25 s steps in intervals of 2θ comprised between 1.5 and 10°.

The functional groups of the samples were examined using Fourier transform infrared spectroscopy (FTIR, model Bruker Vertex70) in the wavenumber range of 4000–400 cm^−1^. Textural properties were determined byN_2_ adsorption–desorption at −196 °C using a surface area analyzer (ASAP 2420, Micromeritics company, Norcross, GA, USA). Both the micropore volume and surface area were obtained using the t-plot method. The surface area was calculated using the multipoint Brunauer–Emmett–Teller (BET) method.

The elemental CHN analysis was carried out with an elemental analyzer (VarioEL cube, Elemental Analyzer system GmbH, Hanau, Germany). The equilibrium pH (eq) was measured using a GLP 21- Crison pHmeter (CRISON INSTRUMENTS, S.A., Barcelona, Spain). The residual metal concentration was determined by ICP-AES (Perkin Elmer, Waltham, MA, USA).

### 2.4. Extraction Protocol

Removal of Cd(II) ions with different solid sorbents was carried out by batch method, and the influence of various parameters, such as contact time, pH, and capacity, was studied. At the end of the predetermined time intervals, the sample was taken out, the supernatant solution was separated from the solid, and the concentration of Cd(II) remaining in the solution was analyzed using atomic absorption spectrometry.

The amount of cadmium trapped by the different solids is expressed as follows:

(1) $$q(mmol\cdot g^{-1})=\frac{{(n}_{0}-{n)}_{mmol}}{m_{g}}$$

q is the sorption capacity of the sorbent materials (mmol/g); n_0_ and n are the mole of the initial and remaining Cd(II) ions in solution, respectively (mmol).

#### 2.4.1. Effect of Contact Time

Kinetic experiments were performed by preparing different tubes containing 0.1 g of solids with 10 mL of a Cd(II) solution (100 mg·L^−1^; i.e., 0.89 × 10^−3^ mmol·L^−1^). The pH values of the samples were adjusted to 5.5 with HNO_3_ or NaOH solution as required. The pH was monitored, and samples were collected at different contact times (0–420) min.

#### 2.4.2. Effect of pH

The sorption of Cd(II) ions was carried out in batch experiments at room temperature (25 °C). A total of 0.1 g of the solid phase was mechanically mixed in polypropylene tubes with 10 mL of aqueous solutions containing cadmium (100 mg·L^−1^, i.e., 0.89 × 10^−3^ mmol·L^−1^) at different pH levels, controlled by the addition of HNO_3_ or NaOH, as required. The tubes were stirred for 60 min, and then the solid phase was separated by high-speed centrifugation.

#### 2.4.3. Kinetic Studies

A kinetic study was undertaken to identify the step which governs Cd(II) adsorption onto the MCM-C and functionalized MCM. For this purpose, four models have been tested: pseudo-first-order, pseudo-second-order, Elovich, and intra-particlediffusion, whose linear forms are given below:

The pseudo-first-order is provided by the following relation [62]:

(2) $$\ln\left(q_{e}-q_{t}\right)=lnqe-k_{1}t$$

where $q_{e}$ and $q_{t}$ (mg·g^−1^) are the amount of Cd(II) adsorbed at equilibrium and after t minutes, respectively, and $k_{1}$ is the rate constant (min^−1^), $q_{e}$ and $k_{1}$ are calculated from the slope and intercept of the linear plot $\ln\left(q_{e}-q_{t}\right)$ versus t, respectively.

The linear form of the pseudo-second-order equation can be written as:

(3) $$t/q_{t}=(1/K_{2}q_{e}^{2})+1/q_{e}t$$

$K_{2}$ (g·mg^−1^·min^−1^) is the equilibrium rate constant. The values of $q_{e}$ and k_2_ are deduced from the plot of $t/q_{t}$ versus t.

The Elovich model has been applied satisfactorily to the chemisorption processes and is expressed by the following relationship:

(4) $$q_{t}=(1/b)lnab+1/blnt$$

The parameters (1/b) and (1/b) ln (ab) rates were obtained from the slope and intercept of the linear plot of qt versus ln t, respectively, a (mg·g^−1^ min^−1^) is the initial adsorption rate, and b (g·mg^−1^) is the desorption constant related to the extent of the surface coverage and activation energy for chemisorption [63]. This equation is often validated for systems where the surface of the adsorbent is heterogeneous.

Intra-particle diffusion model: since the ion exchange process consists of many steps, it is necessary to determine the the rate-controlling step. The most common technique for identifying the rate-controling step is fitting the kinetic data to the intra-particle diffusion model. According to Weber [64], an intra-particle diffusion model is given by the following equation:

(5) $$q_{t}=K_{p}t^{0.5}+C$$

Kp is the intra-particle diffusion coefficient, $q_{t}$ is the amount of metal removed at time t, and C is the intercept.

According to this model, if the plot of qt versus t^0.5^ gives a straight line, then intra-particle diffusion is the rate-controlling step [65]. However, if the data present multi-linear schemes, then two or more steps influence the adsorption process, such as external and intra-particle diffusion [66].

#### 2.4.4. Adsorption Isotherm Studies

The Langmuir and the Freundlich equations were used to describe the experimental sorption isotherm data:

The Langmuir equation [67] is:

(6) $$\frac{C_{e}}{q_{e}}=\frac{1}{K_{L}Q_{max}}+\frac{C_{e}}{Q_{max}}$$

where $K_{L}$ is the equilibrium adsorption coefficient (1/mg), $Q_{max}$ is the maximum adsorption capacity (mg/g), $C_{e}$ is the equilibrium solution concentration (mg/L), and $q_{e}$ is the amount adsorbed at equilibrium (mg/g).

The Freundlich equation [67] is:

(7) $$\;logq_{e}=logK_{F}+\left(\frac{1}{n}\right)logC_{e}$$

where $K_{F}$ and n are the Freundlich sorption constants, indicative of the relative capacity and the sorption intensity, respectively.

## 3. Results and Discussion

### 3.1. Characterization

A previous work described the characterization by XRD, FTIR spectroscopy, and N_2_ sorption at 1–196 °C of MCM-41-C in detail [44].

#### 3.1.1. X-Ray Diffraction Analyses

**Figure 2:**
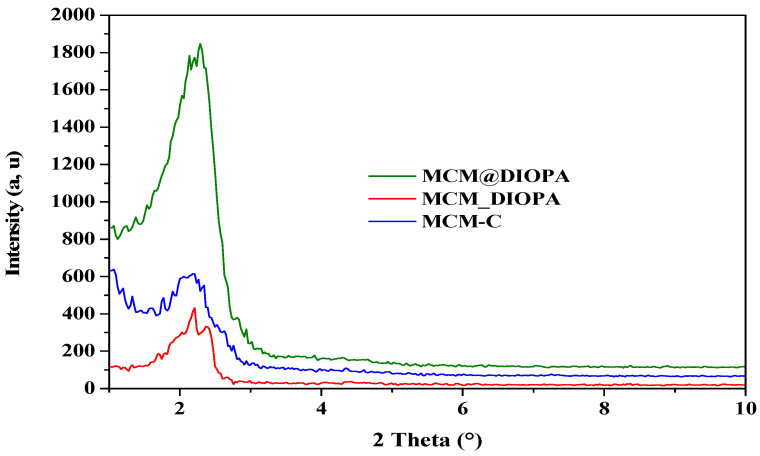
Powder X-ray diffraction patterns of the samples.

The X-ray diffraction patterns of the different solids are illustrated in Figure 2, being typical of those of the mesostructured materials used as support [68,69,70]. Thus, the MCM-C diffractogram shows a peak at 2θ = 2.21°, attributed to the (100) reflection of the hexagonal phase of MCM-41 silica. This diffraction signal is preserved after the incorporation of DIOPA, although the maximum slightly changes (2θ = 2.29°), its intensity decreases, and it is wider. A similar effect was observed by Miloudi et al. [44] after the impregnation of mesostructuredMCM-41 silica with an acylisoxazolone, 3-phenyl-4-benzoyl-5-isoxazolone (HPBI), an acylpyrazolone, 1-phenyl-3-methyl-4-stearoyl-5-pyrazolone (HPMSP), and an organophosphoric acid, di-(2-ethylhexyl)-phosphoric acid (DEHPA).

These diffraction signals correspond to interplanar spacing, d_100_, of 39.94 and 38.55 Å, and the unit cell parameter, a_0_,are 46.13 and 44.53 Å for calcined silica and impregnated calcined silica, respectively.

In the diffractogram of the doped silica, we no longer see the diffraction line at 100 reflection characteristic of the hexagonal mesh. We are, therefore, in the presence of a lamellar structure, which has been observed in previous work by silicas doped with different ligands (HPMSP, DEHPA, and HPBI) [61,71,72]. This can be explained by the presence of a molecule other than the surfactant (DIOPA), introduced during synthesis which decreases the radius of curvature of the micelles. In our case, this diffractogram is characterized by a peak at 2θ = 2.2111, corresponding to 002 reflection, interlaminar spacing, d_002_, is 39.91 Å.

#### 3.1.2. FTIR Spectroscopy Analyses

**Figure 3:**
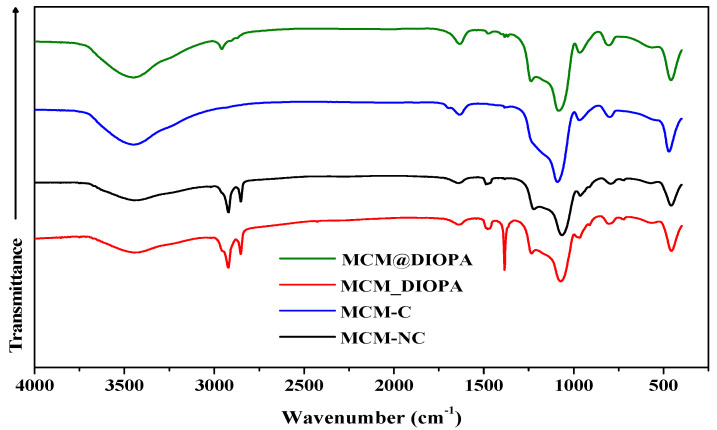
FTIR spectra of the solid samples.

The FTIR spectra (Figure 3) of the three mesoporous materials prepared (MCM-C, MCM_DIOPA, MCM@DIOPA) show the presence of the characteristic bands of the silicate network between 4000–400 cm^−1^. All the solids present the typical absorption bands associated with silica: an intense band between 1080–1090 cm^−1^ associated with the Si–O–Si asymmetrical deformation mode and two weak bands around 460 and 800 cm^−1^ attributed to the corresponding symmetrical deformation mode. A large band is also observed between 3440–3450 cm^−1^ attributed to the stretching vibration of the hydroxyl group (O-H) in silanol groups. Bands between 1630–1640 cm^−1^ are related to the existence of water molecules in the solid support. The functionalized materials showed new bands which would confirm the trapping of the ligand, such as the bands located at 1233 and 1236 cm^−1^ attributed to P-O bonds and the stretching vibrations recorded at 2957 and 2958 cm^−1^ linkedto the C-H bonds of DIOPA. Multiple peaks related to the deformation vibration modes of CH_3_ groups are located between 1369–1480 cm^−1^. These results agree with the literature [22,52,53,54].

#### 3.1.3. N_2_ Adsorption–Desorption Analyses

Textural data were obtained from N_2_ adsorption–desorption at −196 °C (77 K). N_2_ sorption on solids was measured after calcinations at 550 °C for 10 h (before and after functionalization) to remove the surfactant and ligand. The textural properties of the solids used in this study are shown in Table 1.

**Figure 4:**
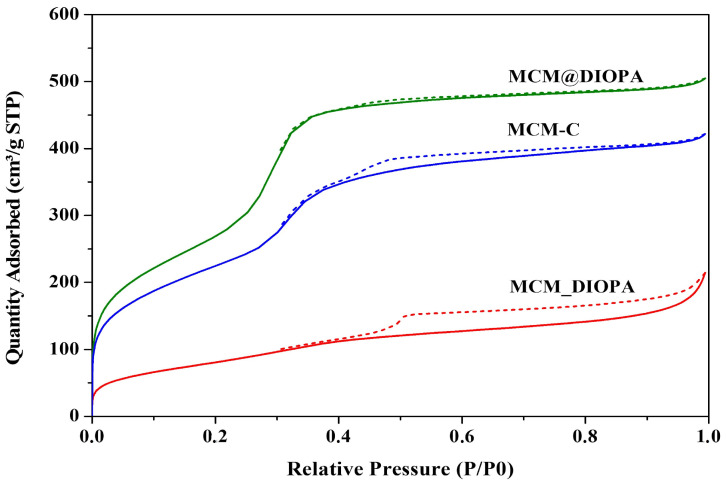
Nitrogen sorption at 77 K.

The N_2_ adsorption–desorption isotherms of MCM-C, MCM_DIOPA, and MCM @DIOPA are of Type IV (Figure 4), according to IUPAC classification, which corresponds to mesoporous solids like MCM-41 [73]. These isotherms comprise three zones: one at low relative pressures P/P0 < 0.2, which is linked to the formation of a liquid nitrogen film on the walls of the pores corresponding to mono-multilayer adsorption. A second zone at intermediate relative pressures (P/P0 between 0.2–0.4) indicates a primary capillary condensation, which is characteristic of mesoporous materials, and a third zone with high relative pressures (P/P0 > 0.4), which is a plateau with a slight inclination attributed to multilayer adsorption. A hysteresis loop is observed between 0.4–0.9, indicating that the pore size is non-uniform and shows pore blockage during desorption. The average pore diameter obtained by the BJH method is between 33.55 and 35.13Å and the total pore volume is between 0.63 and 0.76 cm^3^·g^−1^, which is consistent with mesoporous MCM-41.

MCM@DIOPA shows a specific surface area and pore volume higher than the other two solids (MCM-C, MCM_DIOPA), which could be explained by the enlargement of the pores by the ligand, which would actas a pore expander with its long chain, asdemonstrated for amines by Sayari et al. [74].

#### 3.1.4. Elemental Analysis

An elemental analysis (C, H, N) of the bare and functionalized solids allowed us to calculate the amount of ligand trapped inside the solid as 0.58 and 0.3 mmol/g for MCM@DIOPA and MCM_DIOPA, respectively (Table 2). We note that the quantity of the ligand trapped by the MCM@DIOPA impregnated silica is the same as that obtained by MCM-41 with other ligands [41].

### 3.2. Effect of Contact Time

**Figure 5:**
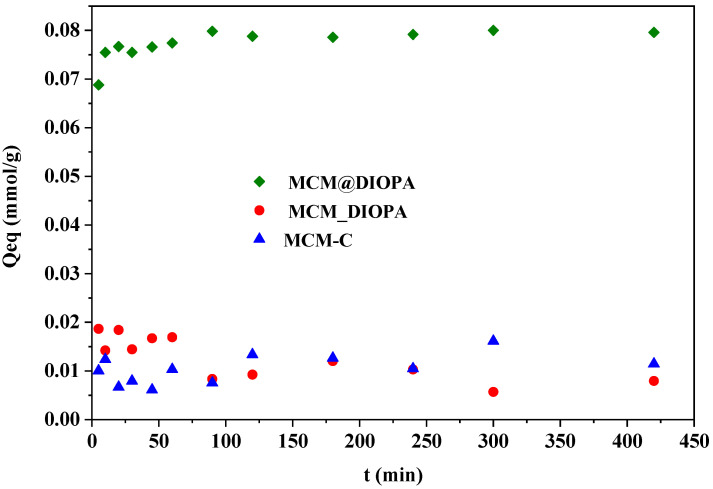
Contact time’s effect on the Cd(II) extraction capacityof MCM-41/DIOPA systems. [Cd(II)] = 100 mg·L^−1^, T = 25 °C, [(Na^+^, H^+^) NO_3_^−^] = 0.1 mol·L^−1^, pH= 5.5.

Contact time is essential for understanding the adsorption mechanisms of the adsorbate. The data presented in Figure 5 show that the maximum adsorption capacity increases with contact time. Based on the obtained data, it can be observed that the adsorption capacity increases in the first 30 min, and, after that, further increases in contact time had no significant influence over the maximum adsorption capacity. Due to that, the optimum contact time for the other experiments was set to 60 min, which was considered to beenough to remove Cd(II) and to reach equilibrium. In the literature, we noticed that the extraction time of cadmium by other materials was longer [75,76]. It is also noted that the quantity of cadmium trapped by MCM_DIOPA is very low compared to that trapped by MCM@DIOPA; this is probably due to the inaccessibility of the ligand to complex the cadmium ions in aqueous solution, which has already been observed [77].

The Cd(II) elimination mechanism was investigated using the pseudo-first-order, pseudo-second-order, Elovich, and intra-particle diffusion models (Table 3 and Table 4).

According to these results, the pseudo-second-order model better fits the experimental data than pseudo-first-order equation since the correlation coefficient is almost equal to 1 and the experimental and theoretical extracted quantities are identical (Table 3). These results imply that the second-order chemisorption mechanism could explain the adsorption of cadmium, which takes place by complexation of the metal cation by the ligands present in the solid phase. This is in agreement with Chafki et al. [78], who studied the adsorption of cadmium on tricalciumphosphate material.

Based on the intra-particle model, the plot of Qt vs. t_1/2_ is linear if intra-particle diffusion is the only rate-controlingstep. In this study, the plot did not pass through the origin, which implies that intra-particle diffusion was not the sole rate-controlingstep. Then, these data demonstrate that other phenomena would control the adsorption process [79]. The results of fitting parameters on these kinetic models are presented in Table 4.

### 3.3. Effect of Equilibrium pH

**Figure 6:**
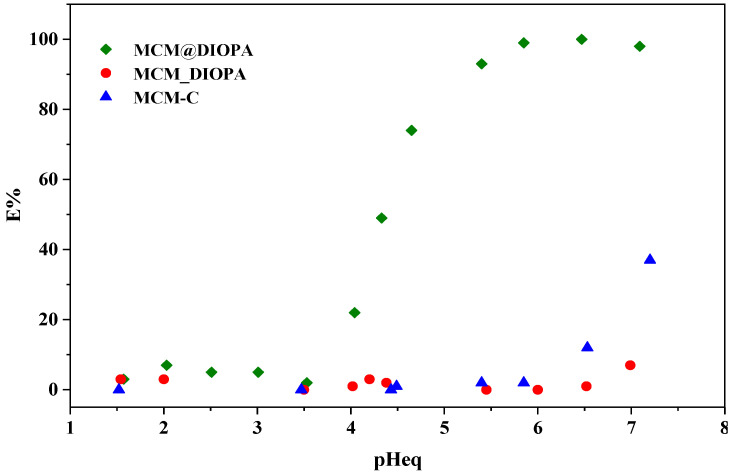
Effect of pH on Cd(II) sorption capacity of MCM-41/DIOPA systems. [Cd(II)] = 100 mg·L^−1^, T = 25 °C, [(Na^+^, H^+^) NO_3_^−^] = 0.1 mol·L^−1^, t = 1 h.

Given that the reactions involved in the adsorption of metal cations generate an exchange of protons, whether those of the silanol sites or of the ligand, it is interesting to look at the influence of pH during adsorption [80]. To study the effect of this parameter on metal sorption by the MCM-C, MCM@DIOPA, and MCM_DIOPA materials, the initial pH was varied between 1.5 and 7 (at a dilute metal concentration of 100 mg·L^−1^). The extractions were carried out under the same conditions in order to compare the properties of the different types of supports. These operational conditions were selected to avoid the precipitation phenomenon. The metal extraction by MCM-C, MCM_DIOPA, and MCM@DIOPA was studied as a function of pHeq (Figure 6).

The results showed that MCM-C and MCM_DIOPA poorly extract cadmium. The low extraction of cadmium by the doped solid can be explained by its inaccessibility to ligand molecules allowing for the complexation of cadmium ions. For MCM@DIOPA, the extraction yield increases with pHeq until reaching a maximum at pHeq = 5.85 [81]. The interaction between the ligand and the MCM can explain this behavior. The complexing sites are more accessible and these are better oriented to bind the cadmium ions.

The decrease of initial pH after extraction can be explained by a cation exchange mechanism proposed by the following reactions:

Cd^2+^ + 3 HL = CdL_2_^–^HL + 2H^+^

This has already been observed during the extraction of cadmium by organophosphorus acids [81,82].

This result is very interesting for the trapping of cadmium ions contained inindustrial discharges, because the extraction pH (5.85) is lower than the pH of extraction of cadmium by other supports and it can be acheved under mild conditions (25 °C) [27,75,81,82].

The rest of the study only concerns impregnated silica since it extracted cadmium better than the other solids.

**Figure 7:**
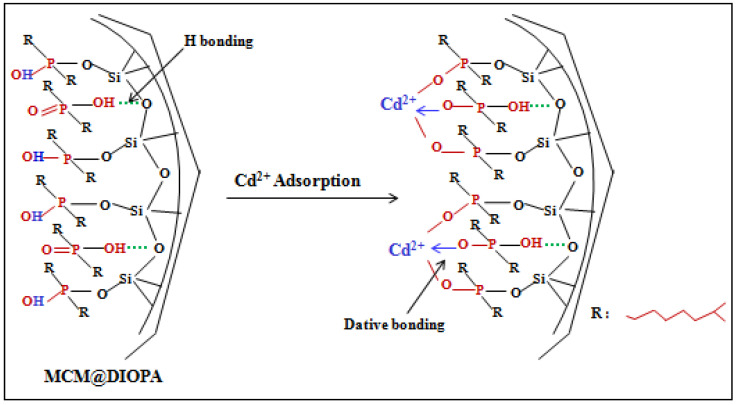
Cadmium (Cd^2+^) adsorption on MCM@DIOPA.

Figure 7 schematizes the interaction between cadmium and the active centers of silica.

### 3.4. Sorption Capacity

**Figure 8:**
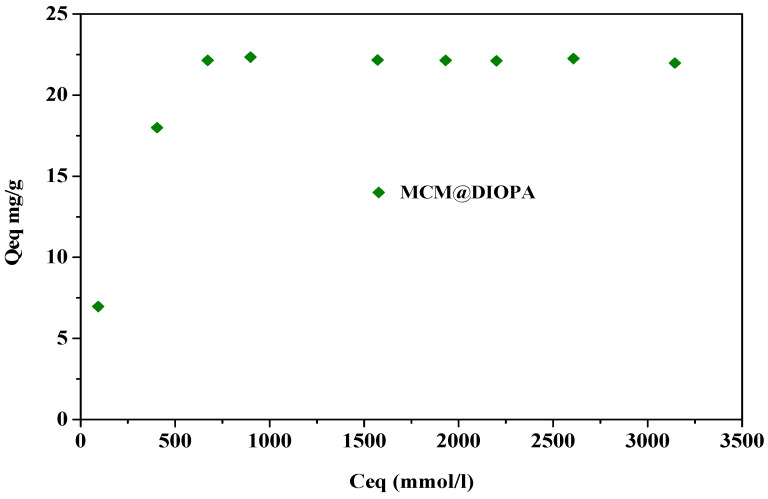
Cadmium sorption capacity of the MCM@DIOPA material. T = 25 °C, [(Na^+^, H^+^) NO_3_^−^] = 0.1 mol·L^−1^, t = 1 h, pH = 5.6.

The adsorption curve is shown in Figure 8 for the MCM@DIOPA solid. The adsorption efficiency increased even at a lower cadmium concentration (500 mmol/L). One plateau was observed with MCM@DIOPA at 22.16 mg/g (0.2 mmol/g). Table 5 presents the sorption capacities of cadmium by different solids. We notice that our solid has a significantly higher sorption capacity at a lower pH [27,75,82,83,84]. The ligand/metal ratios Ls/Ms are calculated where Ls is the total number of free or coordinated DIOPA sites and Ms is the total number of cadmium atoms in the solid. The ratio is equal to 2.9; this can be explained by the formation of the CdL_2_-HL complex. This has already been observed for different metal cations with other solids impregnated with organophosphorus acids [35,81].

In this case, the isotherm obtained is of type L, which is in agreement with the hypothesis put forward in the kinetic study (chemisorption).

### 3.5. Modeling of Adsorption Isotherms

**Figure 9:**
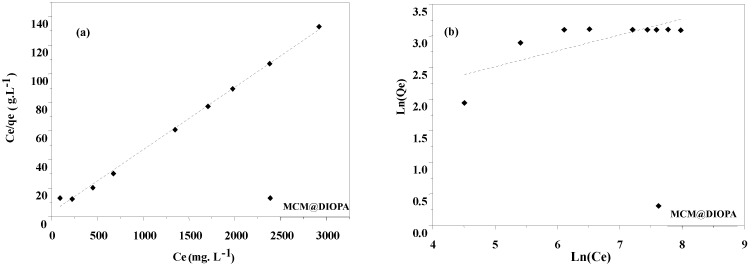
Effect of Cd(II) equilibrium concentration (Ceq) on Cd(II) extraction by MCM@DIOPA, (**a**) Langmuir and (**b**) Freundlich fit lines.

Plots of Langmuir and Freundlich isotherms of Cd(II) extraction by MCM@DIOPA are shown in, Figure 9a,b, respectively. Langmuir and Freundlich constants are summarized in Table 6.

According to the results in Table 6, we see that the Lagmuir model is the most suitable for use. This is in agreement with the results obtained during the kinetic study.

## 4. Conclusions

This study describes cadmium removal from aqueous solution using mesoporous materials functionalized by DIOPA. The adsorption and physicochemical properties of these solids were studied in detail.

Introducing the extractant by different methods (impregnation and doping) will impact their amelioration efficiency for cadmium. In the present work, the different solids were specially conceived to remove cadmium from nitrate solution. Based on the XRD results, the mesostructures of the solids were preserved after functionalization. The data obtained byFTIR spectroscopy and elemental analysis confirm the presence of the extractant in the solid form.

Cadmium extraction by the impregnated solid (MCM@DIOPA) showed better efficiency than that of the doped solid (MCM-DIOPA). The pH solution strongly influenced the metal sorption. The best operating condition was found to be at pH 5.85. A contact time of about 5–30 min was required to achieve equilibrium at the same pH. The kinetic sorption data followed a pseudo-second-order rate equation (psore). The sorption capacity towards cadmium ions was 200 mmol/kg.

To complete this work, it would be judicious to consider the treatment of acid leachates loaded with heavy metals in a future project. The solid MCM41@DIOPA could also be used in the future for analytical pre-concentration and/or depollution of large quantities of water containing low concentrations of heavy metals.

## Figures and Tables

**Table 1:** Textural and structural properties of solids.

Solid	SBET (m^2^g^−1^)	Vp (cm^3^g^−1^)	Dp(Å)	* d_100_/** d_002_ (Å)	a_0_ (Å)
MCM-C	824	0.63	35.13	39.94	46.13
MCM@DIOPA	985	0.76	33.55	38.55	44.53
MCM_DIOPA	298.53	0.26	52.35	39.91	39.91

* Hexagonal structure; ** Lamellar structure.

**Table 2:** Elemental Analysis Of The Materials.

Samples	% C	% H	% N	[HL] mmol/g
MCM-NC **	31.020	6.474	1.843	/
MCM-C	0.090	1.028	0000	/
MCM_DIOPA	36.833	7.155	2.300	0.3,this work
MCM@DIOPA	11.225	2.506	0.029	0.58,this work
MCM-CI-DEHPA MCM-CI-HPBI MCM-CI-HPMSP	/	/	/	0.57 [44] * 0.55 [44]* 0.54 [44] *

* Analysis of washing water by UV–Visible spectroscopy. ** MCM-NC: non calcined silica.

**Table 3:** Kinetic parameters for cadmium adsorption by MCM-41/DIOPA systems: pseudo-first-order andpseudo-second-order models.

	Pseudo-First-Order				Pseudo-Second-Order		
	qe_exp_	qe_th_	k_1_	R^2^	qe_th_	k_2_	R^2^
MCM-C	1.814	2.577	3.10^−4^	0.032	0.082	50.10^−4^	0.9367
MCM_DIOPA	2.09	2.1962	2.10^−4^	0.273	0.812	−15.17	0.9211
MCM@DIOPA	9.46	2.682	41.10^−4^	0.441	9.47	17.02	0.9998

**Table 4:** Kinetic parametersfor cadmium adsorption by MCM-41/DIOPA systems: Elovich and Intra-particule diffusion models.

	Elovich			Intra-Particule Diffusion		
	a (mg/g h)	b (g/mg)	R^2^	Kp (mg/g·h^1/2^)	C (mg/g)	R^2^
MCM-C	0.23283	0.75164	0.09278	0.02978	0.88199	0.19886
MCM_DIOPA	−0.66655	2.63078	0.62055	−0.06966	2.10562	0.64577
MCM@DIOPA	0.49214	8.23153	0.63876	0.04398	8.69185	0.45924

**Table 5:** Adsorption capacicity of cadmium ions on different materials.

Sorbent	pH	Adsorption Capacity (mg·g^−1^)	References
SDS-MCM-41	7.00	8.56	[27]
Bamboo charcoal	8.00	12.08	[75]
Alginate–calcium carbonate	6.00	10.20	[82]
Magnetic nanoparticles	6.00	2.68	[83]
Nanocomposite silica aerogelactivated carbon	6.00	0.38	[84]
MCM@DIOPA	5.85	22.16	This work

**Table 6:** Langmuir and Freundlich constants of Cd(II) adsorption.

	Langmir			Freundlich		
	q_m_ (mg·g^−1^)	K_L_ (L·mg^−1^)	R^2^	K_F_	n	R^2^
MCM@DIOPA	22.89902	0.01356	0.99607	1.46410	3.97377	0.54976

## Data Availability

Data is contained within the article or supplementary material.

## Supplementary Materials

The following supporting information can be downloaded at: https://www.mdpi.com/article/10.3390/molecules29215199/s1, Figure S1: Kinetics of the pseudo-first-order of Cd (II) extraction by MCM-41/DIOPA systems; Figure S2: Kinetic of the pseudo-second-order Cd (II) extraction by MCM-41/DIOPA systems; Figure S3: Elovich model of Cd (II) extraction by MCM-41/DIOPA systems; Figure S4: Kinetics of intraparticle diffusion of Cd (II) extraction.

## Abbreviations

MCM@DIOPA: Calcined and impregnated silica. MCM_DIOPA:Doped silica. MCM-C:Calcined silica.
