# Supplementary material for: Synthesis and Characterization of Mesoporous Materials Functionalized with Phosphinic Acid Ligand and Their Capability to Remove Cd(II)
Source: Molecules. 2024 Nov 2;29(21):5199. doi: 10.3390/molecules29215199 (PMC11547477; doi:10.3390/molecules29215199)
Supplement: Supplementary file 1 [file molecules-29-05199-s001.zip › molecules-3226087-supplementary.pdf]

Kinetics models:

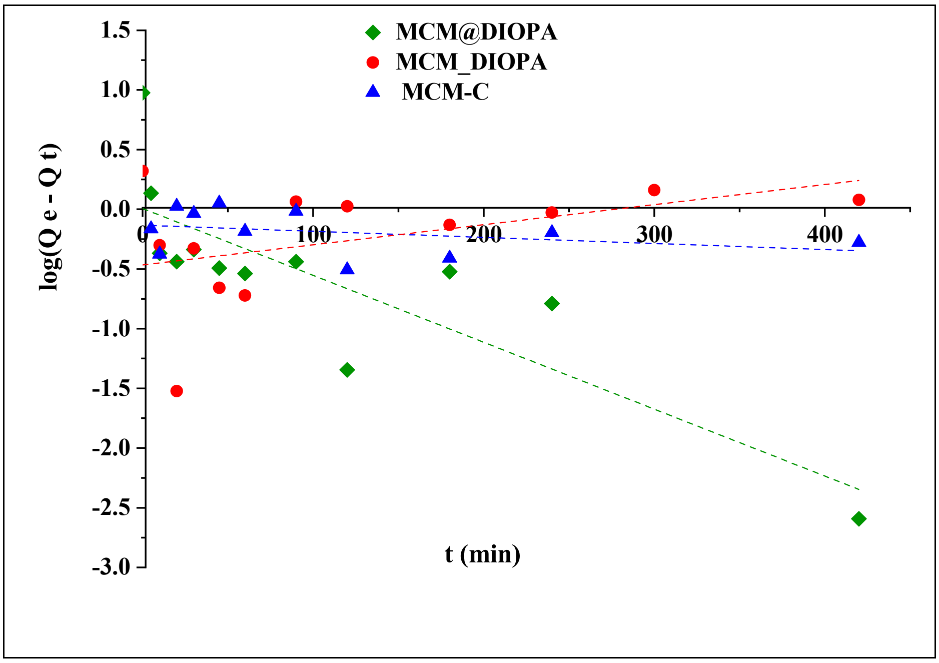

Figure S1. Kinetics of the pseudo-first-order of Cd (II) extraction by MCM-41/DIOPA systems.

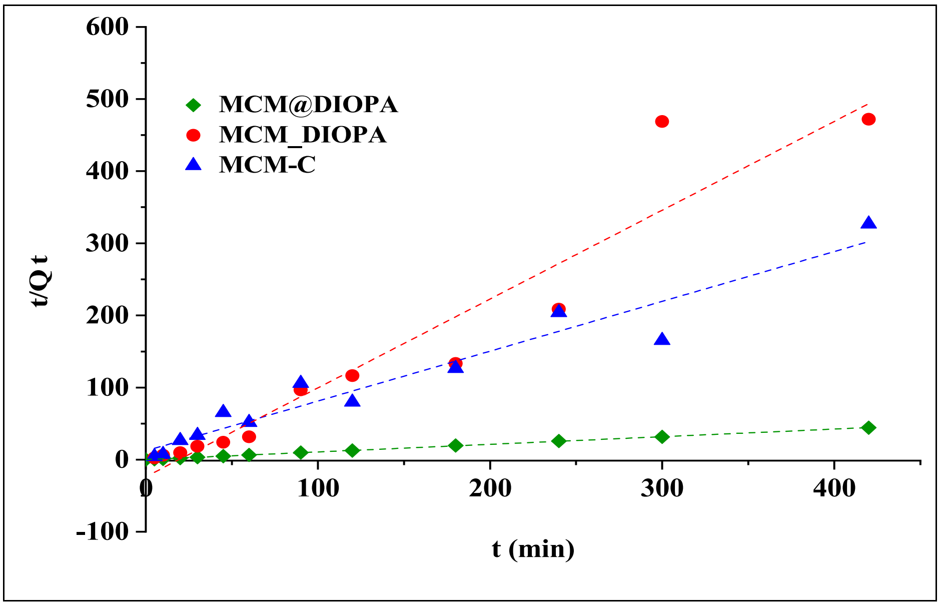

Figure S2. Kinetic of the pseudo-second-order Cd (II) extraction by MCM-41/DIOPA systems.

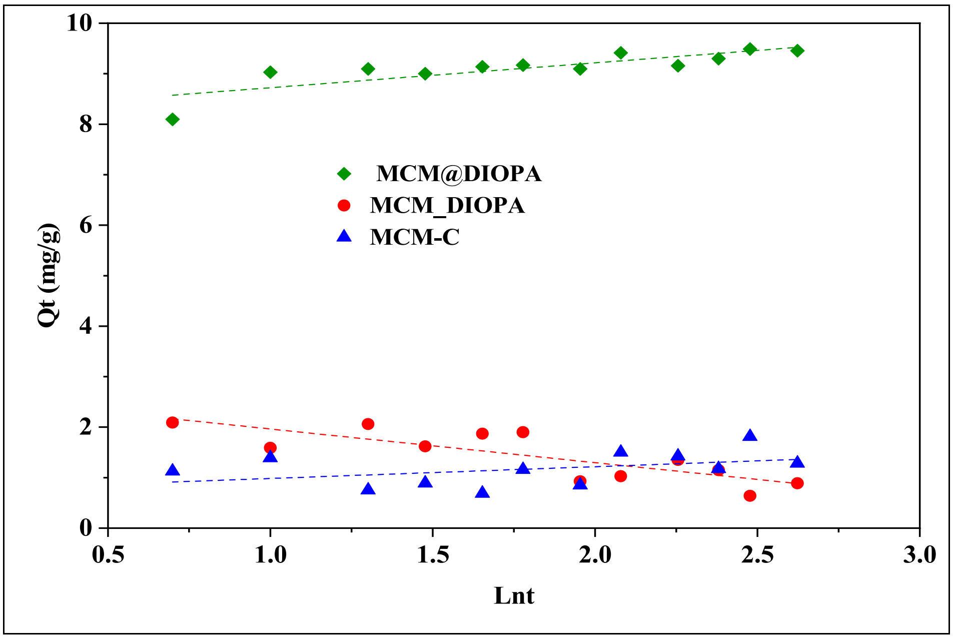

Figure S3. Elovich model of Cd (II) extraction by MCM-41/DIOPA systems

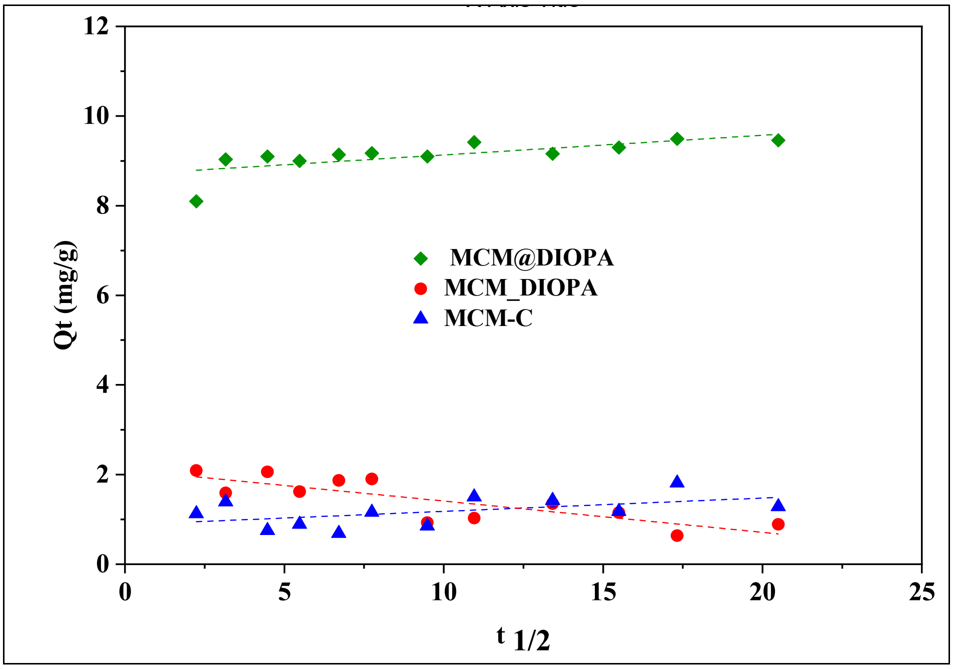

Figure S4. Kinetics of intraparticle diffusion of Cd (II) extraction
